# Patient and Public Involvement in Research Evaluating Integrated Care for People Experiencing Homelessness: Findings From the PHOENIx Community Pharmacy Pilot Randomised‐Controlled Trial

**DOI:** 10.1111/hex.70070

**Published:** 2024-10-18

**Authors:** Andrew McPherson, Vibhu Paudyal, Richard Lowrie, Helena Heath, Jane Moir, Natalie Allen, Nigel Barnes, Hugh Hill, Adnan Araf, Cian Lombard, Steven Ross, Sarah Tearne, Parbir Jagpal, Versha Cheed, Shabana Akhtar, George Provan, Andrea Williamson, Frances S. Mair

**Affiliations:** ^1^ Pharmacy Services, NHS Greater Glasgow and Clyde Scotland UK; ^2^ Florence Nightingale Faculty of Nursing, Midwifery and Palliative Care King's College London London UK; ^3^ School of Pharmacy, College of Medical and Dental Sciences University of Birmingham Birmingham UK; ^4^ Centre for Homelessness and Inclusion Health, School of Health in Social Science University of Edinburgh Edinburgh UK; ^5^ NHS Birmingham and Solihull Mental Health Foundations Trust Birmingham UK; ^6^ SIFA Fireside Birmingham UK; ^7^ Simon Community Scotland Glasgow UK; ^8^ Birmingham Clinical Trials Unit University of Birmingham Birmingham UK; ^9^ General Practice and Primary Care, School of Health and Wellbeing, College of Medical, Veterinary and Life Sciences University of Glasgow Glasgow UK

**Keywords:** health inequality, homelessness, integrated care, patient and public involvement, pharmacist independent prescriber, third sector organisation

## Abstract

**Introduction:**

There is a paucity of research on and a limited understanding of patient and public involvement (PPI) in the context of research in homelessness and, in particular, direct involvement of people with lived and living experience of homelessness (PEH) as expert advisors. We aim to report on outcomes and reflections from lived experience advisory panel (LEAP) meetings and PPI activities, held throughout the study lifecycle of a pilot randomised‐controlled trial (RCT) focused on evaluating integrated health and practical support for PEH.

**Methods:**

Community Pharmacy Homeless Outreach Engagement Non‐medical Independent prescribing Rx (PHOENIx Community Pharmacy RCT) is an integrated health and social care intervention for people experiencing homelessness who present to community pharmacy. Intervention includes weekly support from a pharmacist prescriber and a third sector support worker for up to 6 months. PPI activities undertaken throughout the study were documented, including outcomes of LEAP meetings. Outcome reporting followed Guidance for Reporting Involvement of Patients and the Public 2 Short Form (GRIPP2‐SF).

**Results:**

In total, 17 members were recruited into the LEAP; six meetings (three in two study sites) were held. PPI input was also received through representation from homelessness third sector organisation staff as study co‐applicants and core membership in the trial steering committee. Together, the PPI activities helped shape the study proposal, design of study materials, data analysis and dissemination materials. LEAP panel members offered valuable input via their experience and expertise into the delivery and refinement of interventions. Although longitudinal input was received from some LEAP members, ensuring repeat attendance in the pre‐planned meetings was challenging.

**Conclusion:**

People who face social exclusion and marginalisation can provide highly valuable input as equal partners in co‐design and delivery of interventions seeking to improve their health and well‐being. Fluid membership and flexible methods of seeking and incorporating advice can offer pragmatic approaches to minimising barriers to continued involvement in research.

**Patient or Public Contribution:**

This study reports findings and learning relevant to involvement of people with lived and living experience of homelessness as advisors in a research study. It is important for researchers to offer fluid memberships and use diverse methods to receive input from lived experience members, as traditional PPI methodology may be insufficient to ensure inclusivity. Staff and volunteers from third sector organisations were important PPI partners who bring their experience based on frontline service provision, often as the first port of call for people experiencing severe and multiple disadvantage.

**Trial Registration:**

ISRCTN88146807.

## 1 Background

### 1.1 Introduction

Homelessness can include rooflessness (rough sleeping), houselessness (temporary accommodation in institutions), living in insecure accommodation and living in inadequate housing [1]. In the United Kingdom, people experiencing homelessness (PEH) die at an average age of 45 years, drugs, alcohol and poor mental health being key contributing factors [2]. Homelessness has a bidirectional relationship with poverty, meaning that it can be both an antecedent of poverty and an effect of it [3].

Homelessness is an ongoing challenge in high‐income countries. In the United States, more than half a million people are known to experience homelessness in a given night [4]. A previous survey of the general public conducted in 12 European countries showed that four out of every 100 people in Europe reported having experienced homelessness once in their lives [5]. Latest statistics from England highlight that 112,660 households were housed in temporary accommodation, an annual increase of 12.1%, with mental health and physical health and disability the two most common support needs required to sustain permanent accommodation [6]. A total of 741 and 244 deaths of PEH were recorded in 1 year in England and Scotland, respectively, half of all deaths relating to external (drug misuse, accidents, suicides, assaults) causes [2, 7]. Homelessness forms part of the overlapping triad, including substance use and offending, of severe and multiple disadvantage (SMD) [8]. Access to suitable housing for PEH is perceived as a public health issue [9] due to disparate negative health outcomes [10].

With notable exceptions [11], very few randomised‐controlled trials (RCTs) evaluating interventions for PEH exist. One reason for this lack of RCTs could be that researchers often find it challenging to recruit and particularly retain PEH as study participants in RCTs. A recent review conducted as part of the National Institute for Health and Care Excellence (NICE) guideline on the health and social care of PEH identified the need to involve people with lived experience of homelessness in research [12].

#### 1.1.1 PHOENIx Community Pharmacy Pilot RCT

We carried out a pilot RCT of the Pharmacy Homeless Outreach Engagement Non‐medical Independent prescribing Rx (PHOENIx) intervention for PEH and recruited participants from community pharmacies in Birmingham and Glasgow, United Kingdom. PHOENIx is a complex health and social care intervention with pharmacist independent prescribers plus third sector charity workers operating in tandem. PHOENIx workers aim to see participants at least once weekly via assertive outreach and assess and tackle health, housing, social and practical issues systematically, and informed by what the participant has deemed most important for them at the time. We recruited 100 participants into the study (50 from Birmingham and 50 from Glasgow) and randomised them 1:1 into PHOENIx plus usual care or usual care only. We followed up participants at 3 and 6 months. The study methodology has been reported elsewhere [13].

### 1.2 Patient and Public Involvement in Health and Social Care Research

PPI in health and social care research refers to ‘patients or other people with relevant experience contributing to how research is designed, conducted and disseminated’ [14]. PPI is an active partnership in the research process and is characterised by undertaking research ‘with’ or ‘by’ people who are users of services, rather than ‘to’, ‘about’ or ‘for’ them [15]. Lived Experience Advisory Panels (LEAPs) refer to a group of people who are experts through lived experience and are able to offer advice and support to the research project and on key decisions. LEAP is an increasingly recognised and valuable PPI tool by research funding bodies [16]. LEAP members can bring unique perspectives to the research team, research processes and outcomes of the research project. Rather than just symbolic input, their involvement throughout the research lifecycle is key to derive benefits to the study [17]. Unstable accommodation, ill health and well‐being, including physical, mental health and substance use [18] disorders, in PEH can, however, discourage researchers from seeking their involvement in research as advisors. Little is therefore known with regard to the inclusion of those with living experience of homelessness as partners in research.

Third sector organisations (TSOs), also referred to as voluntary, community and social enterprise organisations [19], are often the first point of support for PEH. TSOs provide food, clothing, housing advice, advocacy and practical support for PEH. TSO staff bring specialist knowledge in terms of social policy and often work in partnership with statutory services such as adult social care and safeguarding of children and vulnerable adults. Staff and volunteers from TSOs can provide important PPI input through their experience of delivering crises care and their positionality with statutory services. There is a scope to utilise TSO expertise and knowledge as advisors in inclusion health research.

The National Institute for Health and Social Care Research (NIHR) recommends PPI input at all stages in the research process [20]. However, PPI input is known to be exercised less when designing a research project [21]. A recent review of co‐production practices in funded applied health research in the United Kingdom showed that only 10 of the 19 studies reported on PPI input on the development or evaluation of the intervention [22]. In part to help identify and reflect the need to involve PPI in services development and evaluation, standards for better public involvement in health and social care research have been devised [23]. Despite these standards, it is acknowledged that such engagement seldom happens [24], including during commissioning processes [25].

The aim of this paper is to report and describe our approach to PPI, including outcomes and reflections from LEAP meetings and PPI activities undertaken as part of the PHOENIx community pharmacy pilot RCT [13].

## 2 Methods

### 2.1 Phoenix Community Pharmacy Pilot RCT

The PHOENIx community pharmacy pilot RCT is a multicentre community pharmacy‐based study, evaluating a complex health and social care intervention for PEH in two cities in the UK (Birmingham and Glasgow). PHOENIx intervention team (NHS pharmacist independent non‐medical prescribers and TSO workers undertaking outreach in community pharmacy, street, homelessness support hubs and other venues) aimed to visit participants at least once weekly via assertive outreach and assess and tackle health, housing, social and practical issues systematically and based on patient priorities.

Participants are adults ≥18 years of age, experiencing homeless using the European Typology of Homelessness and housing exclusion (ETHOS) typology [1] (with the exception of those threatened with homelessness). People living in accommodation with 24 h support, including in‐house medical support, intoxicated or, in the opinion of the researcher, posing a safety risk to others and deemed lacking the capacity to consent as per guidance set out by the Health Research Authority [26] were excluded. Participants were recruited from a total of five community pharmacies. Community pharmacy teams assisted with participant identification and referral to the study researchers, facilitated the use of pharmacy consultation rooms for intervention delivery and supported the study team with follow‐up. The intervention team constituted an NHS pharmacist and a third sector worker who were not part of the community pharmacy team. After consent and collection of baseline data, participants were allocated on a 1:1 basis to either PHOENIx plus usual care or usual care only. We recruited 100 participants into the study. The intervention involved weekly visits from the pharmacist prescriber and third sector support worker, who offered health, housing, practical and social issues for study participants. Participants were followed up at 3‐ and 6‐month intervals. Primary outcome measures for the pilot RCT were recruitment and retention numbers at the 3‐ and 6‐month follow‐up, adherence to the intervention, completed assessments by participants at planned visits, routinely collected emergency department visits and mortality outcomes at the 6‐month follow‐up. A number of secondary outcomes were measured and collated into clinical measures, drug‐ and alcohol‐related measures and social outcomes measures (ISRCTN88146807). The detailed study methodology has been reported elsewhere [13].

### 2.2 PPI Input From Third Sector Organisation (TSO) Staff and Volunteers

The research team built on existing relationships with TSO staff and volunteers through previous research [27] and through our prior commitment to therapeutic engagement and treatment of PEH in TSO drop‐in venues, allowing us to develop trust between TSO staff and volunteers and PHOENIx research staff. Former relationships with TSO staff and volunteers helped us to identify TSO leaders who were interested in and committed to the study as co‐applicants, collaborators and key members of Trial Management Group. Minutes were produced after each meeting, highlighting any specific input from the TSO representative and documenting any advice received.

In total, two staff members representing a TSO each in Birmingham and Glasgow served as study co‐applicants and co‐investigators on the study. Both were included in the trial management group (TMG), which met regularly. A TSO staff member was also part of the trial oversight committee. The committee met bi‐annually, or as required, contingent on the needs of the trial.

### 2.3 LEAP

A PHOENIx LEAP was established to enable a consolidated point of contact for PPI‐related involvement and activities in the study. LEAP members were identified through TSO partners of the study who opportunistically approached people with lived experience of homelessness when they were using the TSO for support and advice. TSO partners initially spoke with service users and asked if they were interested in voicing their views about services for PEH, and if they could commit to the three LEAP meetings. TSO partners approached people they already had a trusting relationship with, making it easier to engage with them, in turn making it easier to build a therapeutic alliance with PHOENIx researchers.

Membership of each LEAP meeting consisted of individuals with lived/living experience of homelessness who were not participants in the PHOENIx Community Pharmacy Study. Meetings took place in private meeting rooms within TSOs. To help make members feel comfortable, tea/coffee, water, soft drinks and food were provided and each member and staff introduced each other. Although researcher approach to LEAP sessions was informal, basic meeting etiquette was encouraged, such as making sure that those who wanted to speak did so, and members and staff listened to them quietly and were courteous to each other. After meetings, members were encouraged to stay behind and speak to staff, if required, in order that they could discuss any problems/issues that had arisen from the meeting.

Terms of reference (TOR) were developed, describing roles, expectations and voluntary contributions, and these were issued to members at the start of each meeting (Supporting Information S1: Appendix 1). Before the opening of meetings, members were asked if they felt comfortable talking about their personal experiences in the presence of others. Members were reassured that the meetings were closed and when reported upon, all comments made by members would be anonymous. The TOR was signed by the LEAP members and researchers. Formal consent was not used as per PPI guidelines, as members acted on an advisory capacity rather than as study participants [28]. Study researchers, third sector support staff and volunteers explained the TOR and answered any questions from the panel members. A study lay summary was also read aloud before each meeting.

LEAP advice was received through iterative meetings held within TSO venues. Reimbursements were paid in the form of £30 shopping vouchers for each meeting as per PPI guidance [29]. Members were also reimbursed for their travel expenses. We used a standardised research impact log [30] to record the outcomes of the LEAP meetings. See Supporting Information S1: Appendix 2.

### 2.4 Reporting

The reporting framework set out by Guidance for Reporting Involvement of Patients and the Public 2 Short‐Form (GRIPP2‐SF) [31] was used to inform the PPI reporting aspect of this research. See Supporting Information S1: Appendix 3.

### 2.5 Ethics Approval

Ethics approval for the PHOENIx Community Pharmacy study was received from Leicester South Research Ethics Committee (22/EM/0119). Governance approvals were obtained from respective research sites.

## 3 Results

PPI input and contributions to the research are described using four overarching themes. These include (1) successfully engage and actively contribute to effective participant follow‐up, (2) help refine an intervention, (3) identify and recommend the most effective approach to delivering services, focussing on what mattered to people, and (4) review dissemination materials and promote trustworthiness and rigour of qualitative data from process evaluation. Impact outcome tables specific to each LEAP meeting describing specific input and learnings have been provided in Supporting Information S1: Appendix 2.

### 3.1 Theme 1: Successfully Engage and Actively Contribute to Effective Participant Follow‐Up

The two TSO members of the TMG provided ongoing advice and support to the study. They advised on the design, intervention components, use of appropriate language, strategies for community engagement, local infrastructure, local policies, links with health and other social care networks and risk management. They were able to liaise with partner agencies to ensure appropriate and satisfactory recruitment and follow‐up. They also advised on the methods that could be used to identify and follow up participants. For example, the use of service databases that existed within TSOs, local authority outreach teams and Department for Work and Pensions (DWP) were recommended to support follow‐up. LEAP members proposed the need to liaise with different agencies, including police and accommodation providers, to find people who go to different cities, and suggested that welfare agencies could also assist with the follow‐up.

### 3.2 Theme 2: Refining the Intervention

Members of LEAP provided useful advice about refining the PHOENIx intervention. For example, they identified patterns of novel psychoactive substance use amongst PEH, including the use of synthetic cannabinoids, such as mamba. Many described having seen deaths in the streets, including within their social network, due to new psychoactive substance use and highlighted the importance of incorporating prevention actions within the PHOENIx intervention, such as the provision of drug education to PEH. Participants also described the widespread use of more customary substance use amongst PEH, including cocaine, ‘street valium’ and pregabalin, and emphasised the important potential educational role for PHOENIx. Members also advised the PHEONIx team to facilitate return to safe spaces for drug consumption, as drug use in temporary accommodation was leading to a prohibition on visitors, which had impacted on the mental health and well‐being of tenants.

LEAP members described that mental health services often do not address patient needs. They mentioned that prescribing decisions often did not involve their views and preferences. They emphasised the importance of using a flexible approach in offering person‐centred care. Members advised that PHOENIx could also help with the overlooked physical and environmental harm caused by rough sleeping. They advised that PHOENIx should facilitate services for exercise, places to shower and freshen up, as many ‘muscular men’ now present as very vulnerable.

### 3.3 Theme 3: Identify and Recommend the Most Effective Approach to Delivering Services, Focussing on What Mattered to People

LEAP members highlighted the importance of offering integrated care and support to PEH. PHOENIx was deemed to have the potential to bridge the gap through appointment support and same‐day referrals. Members stated that PEH value outreach services more than building‐based and traditional GP practices. Members stated a preference for a multifunctional hub where PEH could get their needs addressed in one place. Participants highlighted the need for the services to be more flexible and out with the current ‘9 to 5’ set‐up, appending that crises rarely happen during the working day. Many also felt that addiction and mental health services were not integrated.

LEAP members viewed PHOENIx as a way to integrate addiction and mental health services, undertake physical health screening and minimise the need for people to attend Emergency Departments. PHOENIx was deemed to have the potential to bridge the gap through appointment support and same‐day referrals. Members described their own experiences of having to take extraordinary measures, such as self‐sending to prison and being close to committing suicide because of the lack of support available to them in the past. One member stated that they had 20 care managers in less than a year with Housing First (HF), a policy of unconditional permanent housing first, with support services provided once housing is established, and has now given up on the service.

LEAP members suggested that a lack of procedures and supports for PEH discharged from hospital or prison was an issue that the PHOENIx intervention could address. An example was given of one person discharged from hospital in a gown and sleeping at a bus shelter for 4 days. A member of the public called an ambulance and he was taken to a TSO, not hospital. Several members stated that they went for weeks without a medication prescription because of problems getting to a GP practice or with GP practice registration (no longer registered with a GP on liberation from prison).

Integrated working across sectors was also emphasised in the context of homelessness prevention. For example, members described how removal of children from their family homes by social workers could exacerbate drug use in parents and lead to homelessness.

### 3.4 Theme 4: Review Dissemination Materials and Promote Trustworthiness and Rigour of Qualitative Data

PPI representatives from the TSOs assisted with reviewing dissemination materials, including lay summary and conference abstracts. They also promoted the study through partner networks.

PPI input from LEAP members allowed the research team to ensure the trustworthiness and rigour [32] of the data from qualitative process evaluation. This is an important point because finding people with lived/living experience of homelessness to validate interview data can be challenging. This was done at the final LEAP meetings in both study sites, allowing participants an opportunity to discuss and check thematic headings derived from qualitative interviews with PHOENIx participants and stakeholders [33]. Participants commented on how the experiences of PHOENIx study participants resonated with their own challenges, particularly on the perceived stigma, discrimination in care settings, barriers to timely access of services and nonintegrated nature of services.

LEAP members mentioned that their own perceptions around lack of integration of services resonated with those of participants in the process evaluation part of the study. In particular, retelling traumatic histories when presenting to different services was an unpleasant experience for many. Members perceived that staff from some services were stigmatising and lacked empathy, and therefore highlighted the need for further education and training. Participants wished for the inclusion of practical and social help to relieve boredom in addition to the help on health, housing and practical support. A tailored approach to the many different forms of homelessness was a method that PHOENIx could also adopt. This validates findings from our previous qualitative research [33].

LEAP members described lack of awareness amongst PEH about what services were available to them, and suggested that PHOENIx should be promoted extensively if it is rolled out more widely in the future.

## 4 Discussion

The PHOENIx Community Pharmacy multicentre pilot RCT [13] has allowed the study team to develop and embed best practice in PPI in research in a challenging context. This study adds to the research carried out previously [34, 35], showing that people with lived/living experience of homelessness in the context of a pilot RCT can successfully engage and actively contribute to effective participant follow‐up, help refine an intervention and identify and recommend the most effective approach to delivering services, focusing on what mattered to people. In addition, our experience builds on previous work that suggests that staff and volunteers can offer important PPI contributions through their first‐hand experience in crisis care.

The published literature emphasises the importance of PPI for co‐production and co‐leadership in helping influence healthcare policy reform and practice improvement to reduce health inequalities [36]. However, very few guidelines provide specific recommendations regarding the inclusion of people who have SMD. One previous study involving PEH with problem substance use in Scotland reported that members enjoyed being involved and felt valued, while expressing a preference for face‐to‐face meetings [37]. The perceived value of participating and being members of an advisory group resonated with the views of our own LEAP members.

Challenges described in the literature around PPI involvement include difficulties in ensuring reflexivity [38], such as challenging assumptions, and critical thinking around how this may impact on the research process, and that contributions are representative of the group as a whole [39]. Key experiences shared by LEAP members in relation to barriers of access to health and care services resonate with findings from previous research [40] and validate the process evaluation findings involving perspectives of patients and stakeholders from this study [33].

Integrated approaches to provision of health, housing and practical support such as provided by the PHOENIx model of care were deemed by LEAP members to minimise barriers of access to care. A number of learning points are derived from the PPI activities. For example, some members suggested advertising PHOENIx more widely, helping in turn to inform future study design, particularly around recruitment into the study, ensuring that potential participants who are interested in the study have a chance to take part in it. The way the intervention is delivered, such as tailoring it around specific types of homelessness, such as rough sleeping, is an important aspect that only arose after LEAP members highlighted it. It was thought that PHOENIx could be a conduit between mental health and addictions services, a proposal requiring further scrutiny. One important reflection was that PHOENIx could be used for those liberated from prison, and again, we found that this idea was worthy of further exploration. The study team plans to use the relationships established with LEAP members to help disseminate results of the study, inviting some members to conferences and other research dissemination events.

Paradigmatic participatory health research asserts that those who would be deemed potential participants of the research are the very ones whom we should embrace as contributors in its very co‐creation [41]. Although involving peer researchers and people with lived/living experience of homelessness is rare in trials, it is nevertheless common in other types of research involving PEH. For example, studies utilising qualitative and survey methodologies are increasingly using community researcher models for data collection [42]. Important findings, such as highlighting major obstacles when trying to access mental health support, were obtained when peer researchers carried out interviews with PEH [43]. Peer researchers have also been key to understanding the life experiences of people with SMD in order to gain a better understanding of issues leading to SMD, and for potential intervention opportunities [44]. Peer researchers have carried out qualitative interviews with PEH to establish potential causes of poverty and to identify possible markers of exclusion from services [45]. The Scottish drugs Forum (SDF) carried out seminal research using people in recovery to conduct qualitative interviews with people who use drugs, highlighting significant childhood psychological trauma [46]. SDF along with Healthcare Improvement Scotland and Homeless Network Scotland pioneered an approach using peer researchers, on an understanding that trust is based on shared life experience, helping to acquire more honest and in‐depth results [47]. A further example of peer research involves a study aided by Pathway, a UK homeless peer advocacy charity, resulting in a PPI group helping to inform a future funding proposal for physiotherapy research for PEH [48].

### 4.1 Strengths and Limitations

Strengths include the diverse methods for PPI input used, including people with lived experience and representations from TSOs, providing input into LEAP and trial management committees. Other key strengths include the representation of members with diverse experiences of homelessness, rough sleeping, prison, experiences of the care system, drugs and alcohol and mental health issues. Members appreciated the opportunities available to help improve and refine the nature and development of services, something most members said they had never been asked about before. The use of GRIPP‐2 guidance for involving and reporting PPI activities ensured robustness of our procedure and reporting [31]. Recruitment into the study was supported by expert knowledge of TSO co‐investigators and built on previously established trusting relationships. This in turn aided the building of relationships between researchers and participants. Moreover, TSO co‐investigators helped to provide resources, such as the use of private areas within the TSO, to help recruit, interview and follow up participants.

A particular challenge for the research team was ensuring continued participation of LEAP members in repeat meetings. This was not unexpected and hence fluid membership was offered to the members. In addition, we identified the difficulty for researchers in keeping members engaged for longer than 1 h in a focus group setting. Moreover, note‐taking at LEAP meetings was challenging when recording and subsequent reporting of outcomes.

## 5 Conclusion

This study shows that PPI methods, including TSO staff as co‐applicants to a research project and people with lived and living experience of homelessness, can contribute successfully to a multicentre RCT for PEH. TSO staff are instrumental at introducing potential participants to researchers, and for the use of resources, including a place where participants know, feel safe and are comfortable in. They bring important perspectives from service users and can facilitate recruitment from their service base for research and LEAP participation. People with lived and living experience of homelessness can provide meaningful advice and input as advisors on research studies. PEH value the opportunity to offer contributions to shape the quality and direction of research that is aimed at addressing problems relevant to them. Membership should, however, be flexible and fluid to reflect priorities in the lives of PEH, with methods of input/advice helping to maximise benefits in future research.

## Author Contributions

**Andrew McPherson:** writing–review and editing, writing–original draft, investigation, formal analysis, data curation. **Vibhu Paudyal:** conceptualisation, methodology, supervision, formal analysis, validation, funding acquisition, writing–original draft, writing–review and editing, investigation, resources, project administration, visualisation. **Richard Lowrie:** conceptualisation, funding acquisition, writing–original draft, methodology, validation, formal analysis, supervision, writing–review and editing, project administration, investigation, resources, visualisation. **Helena Heath:** writing–original draft, writing–review and editing, data curation, investigation. **Jane Moir:** writing–review and editing, writing–original draft, investigation. **Natalie Allen:** writing–original draft, writing–review and editing. **Nigel Barnes:** writing–original draft, writing–review and editing, supervision. **Hugh Hill:** writing–original draft, writing–review and editing. **Adnan Araf:** writing–original draft, writing–review and editing. **Cian Lombard:** writing–original draft, writing–review and editing. **Steven Ross:** writing–original draft, writing–review and editing. **Sarah Tearne:** project administration. **Parbir Jagpal:** writing–original draft, writing–review and editing. **Versha Cheed:** formal analysis, writing–original draft, writing–review and editing, data curation, software. **Shabana Akhtar:** writing–original draft, writing–review and editing. **George Provan:** writing–original draft, writing–review and editing. **Andrea Williamson:** writing–original draft, writing–review and editing, supervision. **Frances S. Mair:** writing–original draft, writing–review and editing, supervision.

## Ethics Statement

This study was approved by the East Midlands—Leicester South Research Ethics Committee. Reference number: 22/EM/0119.

## Consent

Terms of Reference was signed by the LEAP members and researchers. As per NIHR PPI guidance, formal consent was not sought, as members acted on an advisory capacity rather than as study participants.

## Conflicts of Interest

The authors declare no conflicts of interest.

## Supporting information

Supporting information.

## Data Availability

Only scientifically sound proposals from appropriately qualified Research Groups will be considered for data sharing. The request will be reviewed by the BCTU Data Sharing Committee in discussion with the Chief Investigators and, where appropriate with any of the following: the Trial Sponsor, the relevant Trial Management Group and independent Trial Oversight Committee. Trial Sponsor: University of Birmingham, Birmingham, B15 2TT, United Kingdom.
