# Supplementary material for: Patient and Public Involvement in Research Evaluating Integrated Care for People Experiencing Homelessness: Findings From the PHOENIx Community Pharmacy Pilot Randomised‐Controlled Trial
Source: Health Expect. 2024 Oct 18;27(5):e70070. doi: 10.1111/hex.70070 (PMC11489131; doi:10.1111/hex.70070)
Supplement: Supplementary file 1 — Supporting information. [file HEX-27-e70070-s001.doc]

**Appendix 1. PHOENIx Community Pharmacy Patient and Public Involvement Group**

**Terms of reference**

This document contains the terms of reference (TOR) for the Patient and Public Involvement (PPI) Group for The PHOENIX CP Trial (Pharmacy Homeless Outreach Engagement Non-medical Independent prescribing Rx community pharmacy-based pilot randomised controlled trial).

PHOENIx CP is funded by the National Institute for Health Research – Health and Social Care Delivery Research Programme (NIHR HS & DR).

PHOENIx is sponsored by the University of Birmingham (UoB).

PHOENIx Co-Chief Investigators (CI) are Dr Vibhu Paudyal [v.Paudyal@bham.ac.uk](mailto:v.Paudyal@bham.ac.uk) and Dr Richard Lowrie

PHOENIx is managed by Birmingham Clinical Trials Unit – University of Birmingham (BCTU), and the Trial Manager is Emily Dixon [e.f.dixon@bham.ac.uk](mailto:e.f.dixon@bham.ac.uk)

The general contact email address is [phoenix@trials.bham.ac.uk](mailto:phoenix@trials.bham.ac.uk) and phone number….(most folk don’t have email access)

**Purpose / role of the group:**

This PPI Group was established in March 2023. Its purpose is to be a centralised contact for Patient and Public Involvement for the PHOENIx CP Trial.

The aims are:

- To enable lay members to advise on the undertaking of the PHOENIx  CP study
- To identify opportunities for the PPI group to improve participant experience in PHOENIx  CP study
- To enable PPI group members to have an active partnership with study researchers
- To advise on the most suitable ways to engage the public, raise awareness of PHOENIx CP and share study outcomes in public domain

**Membership:**

The group will be comprised of individuals who wish to be involved in research but not necessarily as research participants. They may act as a member of a research project steering group, be an advisor for the research team, or help review the project’s literature such as any presentations or publicity coming from the trial. People with lived experience of homelessness can be a PPI representative, unless they are actually taking part as a participant.

**Terms of Reference/ Expectations**

Group are expected to:

- Understand and respect confidentiality, and agree not to discuss, inappropriately, the information provided to members
- Be respectful of the opinions of others and ensure everyone’s right to speak
- Make a reasonable contribution of time to attend meetings
- Make an effort to read through information sent in advance of meetings
- Offer constructive feedback and take an active role within group discussions
- Make honest mileage and expenses claims, using original receipts where possible.
- Inform the organiser prior to meetings if they are unable to attend.
- Memberships will be fluid where there is nonattendance from the PPI member. A new member may be identified to take over.

These terms of reference will be reviewed at the initial meeting and may change over time reflecting the input of panel members. Participants are asked to sign the statement on the following page.

**Meetings**

Adequate information about meetings will be given to group members one week prior to the meeting so they can decide whether to attend. This will include information on the purpose of the event. Participants are under no obligation to attend.

Meetings will be held **3 times during the course of the study**, and they will be in person meetings. The venue will be circulated in adequate time before the meeting.

**Reimbursement**

Members will be reimbursed for their travel expenses and will be offered a £20 voucher compensation for the first 70 minute (1 hour 10 minutes) meeting and £30 for subsequent meetings with a maximum reimbursement of £80 per person over the course of the study. Members are free to accept or decline this compensation.

**PHOENIx PPI Group Declaration for Members**

I confirm I have read and understood the PHOENIx CP Patient and Public Involvement Group Terms of reference V1.0.

I confirm I have been briefed by a PHOENIx CP study member and have had the opportunity to ask any questions.

I undertake to always be aware of the nature and importance of confidentiality and understand that the consequence of any breach associated to me may mean the termination of my membership of this group.

**Full name:**

**Signature:**

**Dated:**Appendix 2. **Lived Experience Advisory Group (LEAP) outcomes**

| **Group number, venue and attendees** | **Discussion** | **Impact (Outcomes)** | **Other comments/actions** |
| --- | --- | --- | --- |
| Birmingham 1 (2^nd^ May 2023) 5 people with living experience of homelessness 1 researcher, 1 third sector charity worker | Discussed group remit and expectations. Discussion surrounded on questions such as how they could help the project; future modes of communications;  How can PHOENIx be improved in anyway going forward;  How can PHOENIx further engage with people who have previous negative experiences about healthcare;  How do PHOENIx team maximise follow up?  What are the dissemination plans | People most needing help are those who can’t register with GP due to lack of IDs,  Mental health services do not address patient needs and they get prescribed what HCPs want rather than what patients need. PHOENIx could change this  Impact of rough sleeping on physical, environmental harm overlooked. PHOENix could help with that  PHOENIx should facilitate services for exercise, places to shower and freshen up as many ‘muscular men’ now very vulnerable.  Mamba is the biggest killer in Birmingham and prevention/education should focus on this. PHOENIx should have prevention messages to members spoken out clearly. The members lost many of their friends to Mamba and not enough being done at the moment. There is no awareness amongst homeless persons about this. Occasional cannabis users taken mamba and have died.  Building relationship, rapport is key.  Services should go out and check people every few hours in the street.  Need to provide travel pass for homeless persons.  Homeless persons lack awareness about what services were available for them. Also, nobody here knew about PHOENIx. They advised the research team for further dissemination of the project. Need big boards in the town areas about all the services available.  Expressed negative experiences about social workers- in looking after children taken care and this sends parents to take up substance and deteriorates metal health  Dissemination: Provide quotes from persons who have used the service and word of mouth to promote such services in the future is the best way. People who have used best speak for the service. | Members felt that 4-5 months is a big gap for another meeting particularly they have so much to contribute to,  Very keen on helping the project  Feel like the researchers are genuinely interested to help people in need.  All meeting members happy to be named in the report.  Next meeting around Sep/Oct 2023 |
| Glasgow 1 (18^th^ May 2023) 5 people with living experience of homelessness, 1 third sector charity worker, 2 researchers, 1 admin worker | Read /discuss TOR & signed consents  All members aware of Phoenix model  Members evaluated intervention  Members commented on intervention and discussed what works, what doesn’t and suggest improvement  Major themes of homelessness- and trauma  Considerations for discussion at subsequent meetings  Keeping in touch | All members agreed that current situation is dire. No Homeless GP practice and suspension of Phoenix intervention will only make life worse for them  Fragmented/disjointed H&SC provision intolerable and unsupportive – at least Phoenix in Hub - accessible  Need for support in one place and with continuity  Frequent change of case workers/care managers with little or no notice  Impact of rough sleeping on physical health/appearance  Stigmatised, neglected – lack of responsibility from H&SC agencies – never felt this from Phoenix  Substandard/illegal accommodation. Health hazard. Fire hazard. Dangerous – don’t feel safe.  Lack of / no communication between CJ and H&SC. E.g. can’t access prescribed services in certain area if banned from area  Members all agreed that Phoenix worked for them – just not enough provision of it  Hungry and dirty = target and vulnerable  Little or no follow up after liberation/rehab – perhaps Phoenix could fit in here?  Little or no access to dental care/podiatry etcetera  Some knowledge/perceived knowledge of legal requirements of care agencies due to involvement with recovery services  Loss of agency/ worth/ worthless – not seen as human being – MH / suicidal  ‘Phoenix should be able to prescribe more’ | Heroin is oot the windae – coke is king  some hostels are open jails  There should be more Phoenix teams across the city – there’s just no enough help anywhere  SCS [Simon Community Scotland] go above and beyond  SCS is my safe space  Ownership of multiple homeless accommodation sites by one person/family – legalities/ethics?  Camaraderie within homeless community – look out for each other when using  Wanted to tell us about own tenancy pros – cook own food, cons – isolation  All members shared traumatic experience and saw drug use as escape  All members enjoyed the session, thanked us for lunch and vouchers and happy to take part in subsequent meetings |
| Birmingham 2 (28^th^ Sep 2023) 5 people with lived experience, 2 researchers | Discussed how members were doing health and wellbeing-wise since last time, discussed study progress with the members.  How could PHOENIx team maximise reach and engagement?  How can people’s engagement in services be maximised?  How could study members’ understanding of prescribed medicines and importance of adherence be emphasised?  How could public support and perceptions of PEH be improved?  What would be most effective intervention for people’s wellbeing? Where should PHOENIx focus? | Members said they were keeping well since last time. One person had to sell their home to stay in hotel. One person is now volunteering to help others on the street as feels they are in a slightly better position than last time.  They advised that services such as PHOENIx be advertised further as people lack awareness.  As in the first meeting people described that many can’t register with GP due to lack of IDs, PHOENIx should help people who are not able to register with a GP.  Pharmacists they have seen in community pharmacies have potential to help but they haven’t helped enough- focused mainly on dispensing. Side effects of antipsychotics (risperidone) and antidepressants (venlaxafine) were not adequately explained to one member  Pharmacists have the potential to help in better ways e.g. check people’s benefits, offer support, advise/refer housing and social care matters  People can walk into pharmacy- this is a huge benefit  One member described adverse experience with GPs and preferred to go to A&E. PHOENIx can help by reaching out to people- need people who care.  Two members said that fortunately they never had ill health (migrants from Poland and Czech Republic)  One member discussed that they never had any health review in four years despite multiple health problems- PHOENIx could change the status quo.  There should be no excuses that there is no funding for services, one member described their own experience of self-sending to prison and nearly committed suicide  Intervention which would be most effective will be to put more money in people’s pocket. One member receives £260 a month in benefits but have to pay £160. Need to focus on wider determinants of health  Many feel that addiction and mental health services not integrated. PHOENIx has the potential to bridge the gap through appointment support and same day referrals.  Members said no one had asked them before about what and how services should be developed. So, commended the study team. | Members keen on continuing to help the project  All meeting members happy to be named in the report.  Next meeting in Spring 2024 |
| Glasgow 2 (24^th^ August 2023) 4 people with lived experience, 12third sector charity worker, 2 researchers, 1 admin worker | Thanked everyone for returning.  Two members recently discharged from hospital and one liberated from prison.  Discussed:  The removal of Hunter St specialist GP and PHOENIx services  Increased demand at Simon Community Scotland (SCS) hub  Drug use/choice/safe space  Inaccessibility of GPs/prescriptions/mental health services  Medication Assisted Treatment (MAT) Standards  Closure of hotels and shortage of homeless beds. Rehab beds  Stabilisation unit | Members state that there should be a procedure of Discharge (prison or hospital) to Crisis centre – then Rehabilitation but the reality is nothing. There is no support. People are being left to get on with it. One participant had been discharged from hospital with hospital gown on and slept in bus shelter for four days. Member of the public called an ambulance and they were taken to SCS. No GP. No Phoenix Team. No-one. The best way to help would be to have an Outreach service and a Hub – where you know to go. With everything you need at some point in the week/month. Basic needs e.g. dentist/optician/podiatrist - they cannot access. Also, to keep assistance with legal/benefits/housing at the hub. All experts by experience state that outreach services are valued more than traditional GP practices because the workers go out of their way to look for them, to engage, to support and actually help. They felt that workers were less formal and more approachable. They felt they understood their issues/problems better because they could see how they were living on the streets. They value a non-judgemental approach.  Some members felt that their GP practice and/or community pharmacy was too far away from where they were staying. They stated that folks need to be on the streets to earn money. If they spend hours trying to get to a GP/Pharmacy miles away – that’s time taken away from their ability to make some money in the city centre. If they didn’t have money (for transport) or were unwell, they would go without prescriptions. Several members stated they had gone weeks without prescriptions for various reasons: their meds had never been prescribed since liberation/discharge or they could not get to the GP practice they’d been registered with. One participant said they were happy with their GP service but had been referred to Mental Health (MH) services and waited 18 months to be contacted. All felt the same frustrations at the lack of access and stated that frustration and anger mean they end up ‘just using again’.  There was a general feeling that Simon Community Scotland (SCS) was much busier now because Hunter St and Phoenix had been pulled. This impacted on simple, everyday issues like accessing a phone/laptop/PC to find out about benefits/keep in touch with family etc. If there are too many people in SCS, there’s no chance of accessing help that day – even for basic, non-specialist assistance. This was causing a great deal of distress.  Coke (cocaine) continues to be widely used. One participant stated ‘you can buy an eighth for £30 now’. Street Valium and Pregabalin also widely used. Members feel that it’s wrong to keep them in their rooms on their own – it’s isolating and unsafe. They ‘use in twos’ and this is impossible in accommodation. Some wanted the return of ‘safe spaces’.  Two members are very active in various groups and recovery services. They mentioned the Medication Assisted Treatment (MAT) Standards and state these standards are not being followed. One stated that their GP had told them ‘you’re not getting benzos – you’re an addict!’  Members stated that there was now a shortage of beds in the city. There was concern that folks were generally being handed sleeping bags – ‘even women wi weans’. Some stated that the availability of rehab beds had been impacted because more were being used privately now.  Several members recalled entering stabilisation unit. They stated that they had to exaggerate lifestyle etc to be able to gain entry to the unit. One participant said that their son – who had been in recovery for weeks, had used again immediately prior to going in as he felt this was necessary to gain admission. Otherwise he felt they wouldn’t believe he needed help. This had caused a great deal of distress. | The toon’s fell apart since Hunter Street [homeless GP service] shut  Keeping people in hotel rooms in isolation is unsafe – it’s no right  There’s nae rooms anywhere – everybody’s getting sleeping bags – even mothers wi weans  I contacted the crisis centre – they said they’d phone me back – aye – 6 weeks later they phoned me back  Pregabalin and Valium – the combination is killin folk!  There’re people in here every day asking for PHOENIx |
| Birmingham 3 (24^th^ April 2024) 4 people with lived experience, 2 researchers | Sense check the themes and quotes from process evaluation  Discuss early findings from the 3 and 6 months follow up  Identify the most important areas of message from participant point of view  Dissemination plans- how should researchers best reach out to people experiencing homelessness to share PHEONIx Community Pharmacy study results.  How do we maximise participant engagement and follow up in the main study?  What services you would LEAP members like to see in future studies?  How do LEAP members feel about attending these meetings and being part of the LEAP?  How will members use LEAP experience in the future?  How do LEAP members feel that people with lived experience can best contribute to research?  Any other advice for the study team? | Members described that PEH lack of awareness about services available (including PHEONIx community pharmacy) and further highlighted the importance of advertising further.  The difficulty in registering at the GPs due to lack of proof of address was again voiced by members  Non-integration of services means that PEH have to share their traumatic experiences each time with several people and it puts people off seeking services. ‘We are parrots to these different services, telling the same thing each time’. PHOENIx have the potential to bridge this gap.  LEAP members suggested having a mother hub model where everything that PEH need including mental health, substance use, practice support, housing assistance, DWP liaison, food and clothing provisions all made available through one place. Members exemplified SIFA is a model very close to this. A nurse comes here on a Tuesday. Churches have been good place for support.  Therapy (CBT?), consultation will be further elements to focus in future PHOENIx.  Navigating to get to drug and alcohol services, mental health services can be very difficult. Someone walking to services can be distracted on the way e.g. to shop lift or come across drug dealers or not have energy to get there. No one in the meeting had a free bus pass. One member said he knew only people on probation eligible for bus pass.  Appreciated the positive attitude from street outreach team from the City Council.  Homelessness can have so many forms and each individual are different and hence tailored approach to care is what PHOENIx should continue to offer.  Described that GP practice for PEH is a good idea and staff try to help as much but there is overcrowding and if you don’t get there too early, likely that you will not be seen.  PEH easily forget appointments due to other life priorities, PHOENIx can bridge this gap. People also may find difficult to control their emotions and can easily be evicted, struck off from the services. To maximise follow up, services need to be tailored to individual person. Liaise with different agencies including police to find people who go to different cities, welfare agencies can help  Members again flagged that future services should address boredom- e.g. trips to Scotland, gardening, cycling, quiz, essay and poem writing, music etc.  What people want from services is empathy, warm energy, caring attitudes. Some have been helpful but not everyone. Members described experience of service providers’ stigma, negative attitudes towards PEH and slamming phones when trying to get through services  People with lived experience need to be part of the service design and development  Sense checked the themes and quotes from the qualitative process evaluation. Members agreed that these resonated with their own experience. They also said they have heard enough about stakeholders describing lack of funding to support services when it comes to implementation of PHOENIx CP. Members said this should not be the excuse.  Members felt valued and felt their voices were being heard and documented. Face to face meetings will be what will work as phone consultations, appointments are not good fit for PEH. Researcher tone, rapport is key, need to show empathy.  Services do not have enough psychological and trauma informed approaches. | Members would contribute happily if there was opportunity in the future.  All meeting members happy to be named in the report. |
| Glasgow 3 (6^th^ February 2024) 4 people with lived experience, 1 third sector charity worker, 2 researchers, 1 admin worker | Thanked everyone for attending and summarised how Patient & Public Involvement (PPI) meeting would help inform development of the Phoenix model.  All members have had contact with Phoenix Team and were happy/keen to share their personal experiences.  Phoenix & Housing First  Interface Third sector worker  Safe space  Buccal Naloxone  What services would you like to see out there? What would help? | Ensured all members had terms of reference/signed consent to participate etc. All advised of Tesco vouchers by way of thanking them all for their participation.  All members expressed their gratitude to the Phoenix Team. One participant was very clear that they would not be here today had it not been for PHOENIx workers. They said that the outreach nature and persistence of CL&SR had got them into crisis centre and helped turn their life around. When all others had given up – CL&SR kept looking for them and supported them where other services had not.  One participant did say ‘why bother with Housing First (HF) – if it works (as Phoenix) leave it be. We all know Phoenix works’. Another participant stated that there was little continuity at Housing First as he has had over 20 Care Managers in less than a year. He has disengaged from their service due to this issue.  As (SCS) advised that she has been supporting three men currently in acute care. One for 5 months and two for over 2 weeks. Previously this has been unheard of for these patients as they usually discharge against medical advice. AS has been visiting them, taking clean clothes etc, supporting from third sector position across acute and primary care whilst assisting in set up of support packages (Benefits/sheltered care/family liaison etc).  One participant talked about the recent opening of the facility. Concerns regarding support for ‘injectors only’. Felt the facility had been rushed and not properly thought through. E.g. there is no ventilation – ‘they should have had decent peers involved to do the job’. Another participant informed us that he will be volunteering at the facility from March.  One participant discussed the recent communication to carry TWO Naloxone injections as one was becoming less and less effective. They had recently encountered someone who had overdosed and they administered four Naloxone injections with little result. They had recently been involved in a presentation by Scottish Drug Forum (SDF) of a prototype of a new product designed to be administered buccally.  A multifunctional hub where you can get all your needs addressed in one place at one time.  More flexible services. Out with 9 to 5. Crisis rarely happens during the working day.  The beauty of Phoenix is the opt-in nature of it. You’re not penalised for not turning up.  The complex needs team are now out of Hunter St and are now in Albion street. | Members all expressed dismay that the Phoenix service/research is still suspended. One participant has a significant wound and recalled how this would have been ‘fixed’ had C&S still be working with them.  Re continuity – participant felt that workers with HF did not afford him the professional courtesy to advise re change of manager, they would just turn up unannounced and he would have to continually repeat his situation to strangers.  Members agreed that a supportive role by third sector who has knowledge of systems and compassionate approach would be beneficial across acute and primary care. Again – peer support could enhance this role. Embed support from admission as opposed to the point of discharge/liberation. One participant was frustrated that a friend was recently liberated with no GP or ADP contact. He said ‘he knows he’ll be back in the jail next week!  Acknowledgement that the facility does reduce harm and is a pilot. Acknowledged how challenging a volunteer post will be for someone in recovery. Wished him well in the post.  Participant understood the pharmacokinetics of the product and how Naloxone injections were becoming less effective against synthetic opioids. Acknowledgement of this specialist knowledge and understanding. Discussion around hazards of using both products but again acknowledgement of their effectiveness in risk reduction.  Discussion around resource. Would everyone understand that their time with such a service would be limited because of the uniqueness of it? Members stated that would be better than nothing at all! They know how the Phoenix model works. Familiar. Accessible.  ‘So now they’re even less accessible’. |

**Appendix 3.** GRIPP2 short form

| **Section and topic** | **Item** | **Reported on page number** |
| --- | --- | --- |
| 1: Aim | Report the aim of PPI in the study | 9 |
| 2: Methods | Provide a clear description of the methods used for PPI in the study | 10 |
| 3: Study results | Outcomes—Report the results of PPI in the study, including both positive and negative outcomes | 14 |
| 4: Discussion and conclusions | Outcomes—Comment on the extent to which PPI influenced the study overall. Describe positive and negative effects | 18 |
| 5: Reflections/critical perspective | Comment critically on the study, reflecting on the things that went well and those that did not, so others can learn from this experience | 21 |
